# *Bifidobacterium bifidum*: A Key Member of the Early Human Gut Microbiota

**DOI:** 10.3390/microorganisms7110544

**Published:** 2019-11-09

**Authors:** Francesca Turroni, Sabrina Duranti, Christian Milani, Gabriele Andrea Lugli, Douwe van Sinderen, Marco Ventura

**Affiliations:** 1 Laboratory of Probiogenomics, Department of Chemistry, Life Sciences and Environmental Sustainability, University of Parma, 43124 Parma, Italy; sabrina.duranti@unipr.it (S.D.); christian.milani@unipr.it (C.M.); gabrieleandrea.lugli@unipr.it (G.A.L.); marco.ventura@unipr.it (M.V.); 2 Microbiome Research Hub, University of Parma, 43124 Parma, Italy; 3 School of Microbiology, University College Cork, T12 YT20 Cork, Ireland; d.vansinderen@ucc.ie; 4 APC Microbiome Institute, University College Cork, T12 YT20 Cork, Ireland

**Keywords:** *Bifidobacterium bifidum*, bifidobacteria, probiotics, genomics, microbiota

## Abstract

Bifidobacteria typically represent the most abundant bacteria of the human gut microbiota in healthy breast-fed infants. Members of the *Bifidobacterium bifidum* species constitute one of the dominant taxa amongst these bifidobacterial communities and have been shown to display notable physiological and genetic features encompassing adhesion to epithelia as well as metabolism of host-derived glycans. In the current review, we discuss current knowledge concerning particular biological characteristics of the *B. bifidum* species that support its specific adaptation to the human gut and their implications in terms of supporting host health.

## 1. General Features of the Genus *Bifidobacterium*

The genus *Bifidobacterium* belongs to the Actinobacteria phylum and this genus together with nine other genera constitute the Bifidobacteriaceae family [1]. Currently, the genus *Bifidobacterium* is comprised of 80 (sub)species, which are distributed across seven different ecological niches, encompassing the Gastro Intestinal Tract (GIT) of humans, non-human mammals, birds, and social insects; waste water; and the oral cavity [2,3]. Remarkably, these ecological origins may represent the biological niche that is common to all these habitats, which is characterized by the fact that a large number of bifidobacterial hosts are submitting their child to parental care. Thus, their ecological origins are perhaps enabled by maternal inheritance of bifidobacterial cells. Interestingly, this supposition has recently been corroborated by the mapping of (near) identical bifidobacterial strains in mothers and their corresponding children (see below) [4,5].

A small number of bifidobacterial (sub)species, such as *Bifidobacterium pseudolongum*, *Bifidobacterium adolescentis*, *Bifidobacterium pseudocatenulatum*, and *Bifidobacterium bifidum*, have been isolated from various animal/mammalian hosts and for this reason are acknowledged as cosmopolitan bifidobacterial taxa [6]. In contrast, other taxa like *Bifidobacterium breve* appear to be much less widely distributed, perhaps due to adaptative behavior that is host-specific [6]. Among those bifidobacteria that are found in primates, certain bifidobacterial species are commonly identified in adults, such as *B. adolescentis* and *B. catenulatum*, while others, like *B. bifidum*, *B. breve*, and *B. longum* subsp. *infantis*, are more typically found in the fecal samples from breast-fed infants [7]. However, there does not seem to be an absolute infant versus adult division of bifidobacterial (sub)species. Such findings make sense from the perspective of vertical transmission of bifidobacterial species from mother to child, which also encompasses adult-type species like *B. adolescentis* [8,9]. The different preponderance of one species over another in the adult/newborn GIT is influenced by the different composition of the intestinal microbiota in terms of complexity, which in turn is very much determined by host diet [10].

## 2. Bifidobacterial Communities of the Human Gut

Bifidobacteria rapidly colonize the gut of infants within the first weeks following birth, a phenomenon that is believed to be driven in no small part by the bifidogenic activities of specific mother milk-derived oligosaccharides, commonly referred to as Human Milk Oligosaccharides (HMOs). Metagenomic-based analyses revealed a high abundance of a small number of bifidobacterial species such as *B. breve*, followed by “adult-type” bifidobacterial taxa such as *B. longum* and *B. adolescentis* [11]. The level of bifidobacterial abundance in the human gut drops with aging, even if microbial profiling experiments that are based on FISH and metagenomic analyses have predicted that their relative load in the adult large intestine is about 4.3 ± 4.4% of total microorganisms [12,13].

In recent years, several metagenomics as well as culture-dependent investigations of the human gut have allowed a detailed dissection of the bifidobacterial biodiversity that is present in this environment [6,7,14,15,16]. Notably, such analyses revealed that the most abundant and prevalent bifidobacterial taxa existing in the human colon are strains of *B. breve, B. bifidum, B. longum*, *B. adolescentis, B. pseudolongum*, *B. pseudocatenulatum*, and *B. animalis* subsp. *lactis*. These studies have highlighted that the varying composition of bifidobacterial gut communities in different individuals underline both an inter-subject and an intra-subject variability, which is in agreement with the large inter-variability of the overall intestinal microbiota as described previously [12,17].

Recently, detailed cataloguing of bifidobacterial communities that are present during infancy has been performed through the sequencing of the Internally Transcribed Spacer (ITS) region of the bifidobacterial rRNA locus, which when compared to the 16S rRNA gene, allows a substantially higher taxonomic resolution [18]. In this study, the Hierarchical clustering that was built on bifidobacterial community profiles that are present in infant stool samples highlighted the occurrence of four bifidobacterial groups, i.e., bifidotypes, characterized by specific, commonly co-occurring taxa such as *B. breve*, *B. bifidum*, *B. adolescentis*, or *B. longum* [19]. In this context, *B. bifidum* was demonstrated to be an important taxon among these bifidotypes in which its occurrence correlates with numerous other bifidobacterial species that were recovered from infant fecal samples.

Interestingly, a study that compared the fecal microbiota composition of infants that were fed with bovine milk formula or with breast milk by 16S rRNA gene microbial profiling and qPCR showed that both *B. breve* and *B. bifidum* abundance was greater in the feces of breast milk-fed infants than in that of formula-fed infants [20]. Furthermore, it was observed that samples with a higher load of bifidobacteria are characterized by the presence of *B. bifidum* at greater than 10% of the total bifidobacterial population [20]. The link between the highest abundance of total bifidobacteria with appreciable *B. bifidum* populations in the stools of breast milk-fed babies suggests that trophic factors play a role in determining this association [20].

**Figure 1:**
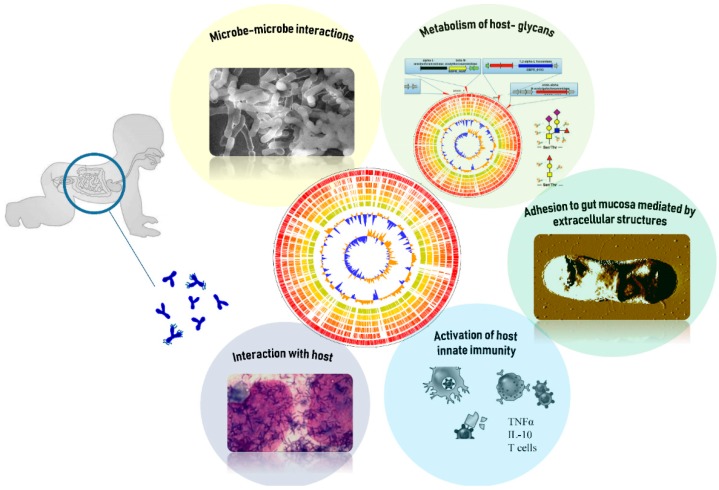
Schematic representation of the main properties exerted by *B. bifidum* in the human gut. Specifically, there is much evidence in the literature that shows that *B. bifidum* is able to interact with the host and with other members of gut microbiota by different mechanisms, activate the host immunity, adhere to gut mucosa with its extracellular structures, and metabolize host glycans, such as mucin. See the main text for further explanation.

In addition, bifidobacterial communities that are present in milk samples frequently contain strains of *B. bifidum*, suggesting that milk represents a key vector for vertical transmission of bifidobacteria [19,21] (Figure 1).

## 3. Maternal Inheritance of Bifidobacteria

In recent years, bifidobacteria have been shown to be subject to mother–infant transfer via a vertical transmission route that seems to be operational both in human beings [4,22] as well as in other mammalian species [6]. The most frequently shared bifidobacterial species between mothers and their corresponding children are strains of *B. bifidum* and *B. breve*, which were displayed to persist in the human gut for up to one year of life [5]. Similar results were found in another study that aimed at tracing maternally inherited bifidobacterial strains [22]. These investigations involved a complex set of analyses such as culturomics, shotgun metagenomics, and ITS bifidobacterial profiling experiments [18,23,24,25] and resulted in the identification of *B. longum* subsp. *longum* BLOI2 and *B. breve* BBRI4, which were isolated in two mother–infant couples and were also shown to be maintained in the intestine of babies until six months of age [22]. Similarly, a detailed cataloguing of bifidobacterial communities in 25 mother–offspring couples based on ITS bifidobacterial profiling trials followed by cultivation experiments identified several bifidobacterial strains that occur in the gut of both the mother and her child and of which are also present in the matching human milk sample [14]. Such findings are corroborated by a similar situation in other primates and certain mammals, where bifidobacteria were displayed to be vertically inherited from the mother to her newborn and where the mother’s milk appears to constitute a key vector that drives such events [6]. Even if the intestinal establishment of bifidobacteria is believed to be (partially) maternally determined, some studies postulate that their acquisition occurs during pregnancy [21]. 

The precise biological/evolutionary consequences of horizontal transfer are still obscure, although one may argue that according to the holobiont concept [21], the first microbial colonizers, which include bifidobacteria, exploit crucial physiological/immunological/metabolic functions in driving host development. Another important speculation that is linked to the formation of the infant gut microbiota is that bifidobacteria, in spite of their drop in abundance upon the weaning stage, carry on after their initial transmission to the infant intestine and are then preserved, though perhaps at very low abundances, in the large intestine of the adult to ultimately be vectored to the following generation [22]. The vertical transmission events of bifidobacteria may thus represent the consequence of long-term co-evolution between bifidobacteria and their mammalian hosts.

## 4. Genomics Features of the *B. bifidum* Species

Many bifidobacterial species have had their genomes sequenced in recent years, in particular those that are exploited by the food industry as probiotic microorganisms [21]. In this context, genome sequencing was performed for multiple strains belonging to the species *B. animalis* subsp. *lactis* [26,27], *B. breve* [28], and *B. longum* subsp. *longum* [28]. Similarly, various strains harboring the *B. bifidum* taxon have been subjected to whole genome sequencing [29], allowing comparative genomic analyses, which has shown a closed pan-genome structure. Such in silico analyses uncovered specific genetic strategies, allowing members of the *B. bifidum* species to establish and persevere in the human gut, for example thanks to the synthesis of different types of pili [30,31] or through metabolic abilities pertaining to host-derived glycans [29,32].

The NCBI-deposited reference genome of the *B. bifidum* species is that of the infant stool isolate PRL2010 [32], which was sequenced and published in 2010. A functional classification of the genes occuring in *B. bifidum* chromosomes following the Cluster of Orthologous Genes (COG) families revealed that a large proportion (approximately 14%) are ascribed to the COG family of carbohydrate metabolism and transport [33,34].

## 5. The Glycobiome of *B. bifidum* Species

The glycobiome represents the overall genes that are expected to be responsible for the carbohydrate metabolism and in the *B. bifidum* species, consists of more than 3000 genes that encode putative carbohydrate-active enzymes, comprising glycosyl hydrolases (GHs), glycosyl transferases (GTs), and carbohydrate esterases (CEs). In particular, according to the Carbohydrate Active Enzymes (CAZy) database [35], there are many genes coding members of family GH13, which are largely identified in bifidobacterial chromosomes, commonly for those bifidobacterial strains that are identified from the intestine of mammals. GH13 are common of bacteria and are responsible for the breakdown of an extensive range of glycans, involving plant-derived complex carbohydrates like starch and correlated substrates (e.g., amylose and amylopectin and/or (cyclo)maltodextrins), trehalose, stachyose, raffinose, and melibiose [33]. Interestingly, the *B. bifidum* glycobiome also includes members of GH families that are crucial in host glycan metabolisms, like those harboring GH33 and GH34, representing exo-sialidases, GH29, and GH95, which characterize fucosidases, and GH20 involving hexosaminidase and lacto-N-biosidase activities, which are genes that are predicted to be responsible for the metabolism of host-glycans (see below) [34]. In this context, a comparative genomic analysis based on 15 different strains highlighted the presence of a conserved gene set that appears to uniquely occur in *B. bifidum* strains [29]. This gene set represents core genome sequences encoding various GHs that are specifically involved in mucin breakdown, which is indicative of a nutritional acquisition strategy targeting host-derived glycans [29].

Furthermore, an exhaustive analysis of the genetic backgrounds for carbohydrate uptake in *B. bifidum* species revealed that this taxon contains a relatively small number of genes coding for carbohydrate carriers when associated with other bifidobacterial taxa that are present in the infant intestine, like *B. breve*, *B. longum* subsp. *longum*, and *B. longum* subsp. *infantis* [7,36]. In fact, only 25 genes are estimated to synthetize carrier systems in the *B. bifidum* PRL2010 chromosome and be committed to glycan uptake. However, the other human gut bifidobacterial chromosomes are predicted to encompass between 35 and 68 such genes, as characterized by *B. adolescentis* ATCC 15703 and *B. longum* subsp. *infantis* ATCC 15697, respectively. This suggests that carbohydrate metabolism in *B. bifidum* taxon is limited to a reasonably low amount of carbohydrates.

## 6. The Ability of *B. bifidum* to Metabolize Host-Derived Glycans

The main carbohydrates that are produced by human beings, i.e., host-derived glycans, which may represent a carbon source for bifidobacteria, are mucin and HMOs. Notably, these sugars are found in the gut in high amounts during two different stages of human life, i.e., the infant period (HMOs) and the adult life (mucin). Interestingly, the *B. bifidum* taxon is present during both of these life stages, though its prevalence and abundance are higher during infancy. However, we may argue that its occurrence in the adult gut is supported by the action of glycan substrates such as mucin (see below).

Mucin is a glycoprotein that constitutes the key component of the mucus gel coating, which covers the epithelial surface of the human GIT [37]. The key glycan monomers that were identified in this glycoprotein encompass *N*-acetylglucosamine, *N*-acetylgalactosamine, fucose, galactose, and sialic acid [38]. Notably, *B. bifidum* species is the only member among all the recognized *Bifidobacterium* (sub)species that is capable of growth by means of mucin metabolism [32,39,40].

As mentioned before, a large portion of the predicted GH-encoding genes that were identified in the *B. bifidum* PRL2010 genome represent enzymes that are estimated to be associated with the breakdown of mucin-derived oligosaccharides, most of which are exclusively occurring in the *B. bifidum* genome [29]. In addition, according to the CAZy database [35], the *B. bifidum* PRL2010 chromosome encompasses members of two carbohydrate-binding module (CBM) families, i.e., CBM32 and CBM51, which are predicted to assist in binding to carbohydrates that are encountered in the mucin core structure.

Further information on how *B. bifidum* metabolizes mucin has been obtained from functional genomic approaches, including proteomics- as well as transcriptomics-based analyses [32]. The main *B. bifidum* enzymes that are responsible for mucin breakdown include extracellular enzymes, like putative exo-α-sialidases, as well as a predicted 1,2-α-*L*-fucosidase and 1,3/4-α-*L*-fucosidase, and a putative cell wall-anchored endo-α-*N*-acetylgalactosaminidase [41,42,43]. Further enzymes that are predicted to be involved in mucin metabolism encompass *N*-acetyl-β-hexosaminidases and β-galactosidases. The mucin-breakdown features of *B. bifidum* species is also supported by the occurrence of glycan carriers harboring several families, like the ATP-binding cassette (ABC-type), phosphoenolpyruvate phosphotransferase system (PEP-PTS), and major facilitator superfamily (MFS).

Specifically, the chromosome of *B. bifidum* PRL2010 contains a DNA region encompassing eight genes that are predicted to code for enzymes responsible for the hydrolysis of galacto-*N*-biose, which constitutes one of the key structures of mucin-oligosaccharides. The possible metabolic scenario employed by *B. bifidum* PRL2010 involves the action of extracellular enzymes such as exo-α-sialidases and 1,2-α-/α-1,3/4-*L*-fucosidases that are responsible for de-sialylidation and de-fucosylation, respectively, thus allowing *B. bifidum* access to mucin-derived galacto-*N*-biose [42]. This mucin-derived substrate is then further metabolized by other enzymes coded by PRL2010 like lacto-*N*-biosidase and endo-α-*N*-acetylgalactosaminidase [42,44,45].

Comparative genomic analyses encompassing other public accessible *B. bifidum* chromosomes showed that the estimated genetic background that is responsible for mucin metabolism is well conserved in this bifidobacterial taxon [29].

Mucin breakdown is predicted to diminish the mucin layer and, therefore, decrease the defensive barrier of the intestinal mucosa, rendering this activity as a potentially unwanted occurrence. However, one may also argue that mucin breakdown has evolved as a “host-settler mechanism”. In fact, mucin synthesis in the gut typically starts upon delivery, then occurs for several months and arrives at its mature level at about one year of life [46]. Fascinatingly, mucin metabolism as performed by *B. bifidum* could activate enhanced production of mucin, thus enhancing the depth of the mucus layer wrapping the mucosa and, thus, strengthening the epithelial barrier function [47].

## 7. The Capability of *B. bifidum* to Interact with its Host by Extracellular Structures

Commensal gut bacteria are known to establish a cross-talk with the human host through various structures including pili, nanotubes, capsular structures, S-layers proteins, and flagella [48,49,50,51]. Interestingly, many of these structures are also used to drive the microbe–microbe dialogue occurring between the different players of the human gut microbiota, comprising bifidobacterial strains [52].

Recently, the genetic variety of predicted sortase-dependent pili of bifidobacteria was assessed, allowing the construction of a bifidobacterial sortase-dependent fimbriome database [31]. Interestingly, the identified bifidobacterial fimbrione was shown to be highly variable among the various bifidobacterial (sub)species, possibly as a result of horizontal gene transfer events [31]. Transcriptome experiments and binding assays involving different substrates has demonstrated the importance of bifidobacterial pili in modulating several adhesion capabilities of bifidobacteria to glycans and to extracellular matrix proteins [31], thus corroborating the ecological fitness of bifidobacteria in the human intestine.

Amongst the fimbriome of members of the *Bifidobacterium* genus, the best experimentally and functionally characterized sortase-dependent pili are those of *B. bifidum* PRL2010 [30,53,54]. Such extracellular structures are composed of major and minor subunits, which are covalently assembled through the action of a sortase. These proteins are typically encoded by three adjacent genes, referred to as a sortase-dependent pilus locus. The genome of *B. bifidum* PRL2010 includes three distinct sortase-dependent pilus loci of which only two were predicted to be genetically intact, while the third appears to be non-functional due to a frameshift within the coding region of the gene encoding major pilus subunit [55].

Microarray-mediated transcriptome analysis of *B. bifidum* PRL2010 following murine colonization and following interaction with human cell lines revealed a significant increase in the transcription of the genes including two sortase-dependent pili, named *pil2* and *pil3* [55]. Heterologous expression of *pil2* and *pil3* in the non-piliated *Lactococcus lactis* demonstrated that both pili are promoting adhesion to human gut mucosa by extracellular matrix (ECM) proteins. ECM de-glycosylation triggered a drastic drop in PRL2010 pilus-based binding ability compared to untreated ECM [55], suggesting that N- and/or O-linked glycoproteins are involved in the adhesion of *B. bifidum* PRL2010 pili to ECM.

Recombinant piliated *L. lactis* cells were also displayed to induce an increased tumor necrosis factor alpha (TNFα) response following administration to mice when associated with their non-piliated parent, suggesting that PRL2010 sortase-dependent pili also elicit immunomodulatory activity [55]. The biological significance of this finding may be that triggering TNFα production by pili synthetized by *B. bifidum* PRL2010 represents a cross-talk signal by an early colonizer of the human gut [7,14,53,56]. In this framework, it is worth mentioning that TNFα superfamily members are not only connected to the manifestation of inflammatory diseases [57], but also exert a key role in the rejection of tumors and the response to infections [58,59]. Furthermore, TNFα induction may be crucial for the establishment of cross-talk among immune cells and commensal bacteria without causing inflammation or other harmful effects [60].

In addition, sortase-dependent pili of PRL2010 were shown to promote self-aggregation as well as aggregation of PRL2010 cells with cells of other gut microorganisms including bifidobacteria and lactobacilli [55,61]. This suggests that these extracellular proteins are responsible for the generation of macro-colonies or biofilm in the environment.

Interestingly, the *B. bifidum* species is the only *Bifidobacterium* taxon without capsular polysaccharides genes which seems to be absent due to a recent deletion event [62].

Nevertheless, a small number of isolates of the *B. bifidum* species have recently been shown to produce a complex mixture of polysaccharides, i.e., a negatively charged phospho-glycero-β-galactofuranan (PGβG), four neutral polysaccharides being β-(1-6)-glucan, β-(1-4)-galactan, β-(1-6)-galactan and β-galactofuranan and finally, starch. Interestingly, these two fractions were shown to exert distinct immune responses when assayed on dendritic cells. PGβG increased pro-inflammatory immune responses by enhancing the levels of TNFα, whereas CSGG induced immunosuppressive regulatory T cells and interleukin-10 [63].

Many enteric bacteria possess the ability to transform bile salts biochemically through the deconjugation of bile salts via the action of a Bile Salt Hydrolase (BSH). The detergent action of bile salts helps in the reduction of intestinal absorption and increased fecal elimination [64], with an overall hypolipidemic in vivo effect [65]. Recent screening analyses revealed that BSH activity is widely distributed in many bifidobacterial species (i.e., *B. longum* subsp. *longum*, *B. longum* subsp. *infantis*, *B. animalis*, and *B. bifidum*) [66,67]. *B. bifidum* PRL2010 shows noteworthy BSH action on conjugated secondary bile salts (glycodeoxycholate and taurodeoxycholate) [68,69] in line with what has been observed in other *Bifidobacterium* species, such as *B. animalis* and *B. longum* [70]. The importance of this enzyme for microorganisms is also so far not well characterized. In fact, various suppositions have been suggested, but the specific biological meaning of bile salt hydrolases at this time remains unidentified. It has been theorized that the conjugation of bile salts may be a tool that bacteria use in order to produce bile detoxication and thus, these enzymes may exert a key function in microbial bile tolerance and survival in the human gut [71].

## 8. The Ecological Role of *B. bifidum* Species in the Infant Gut Microbiota

As described above, bifidobacteria are amongst the first microbial colonizers of the infant gut [21]. Upon delivery, the gut environment is subject to massive bacterial colonization, in particular bifidobacteria, which is acquired through vertical transmission involving direct mother–baby contact at birth and is supported through breastfeeding [21]. Notably, under “non-natural” conditions, such as a caesarian delivery or baby feeding with reconstituted or formula milk, the newborn gut may undergo rapid colonization by environmental microorganisms that normally do not occur at that stage or in such abundance, which may provoke long lasting health effects on the host [21]. Supplementation of health-promoting bacteria with, for example, bifidobacteria that are typically found in the infant gut during this very critical window of time may be crucial in terms of preventing the establishment of an aberrant microbiota with its associated negative health implications.

In this context, it has been shown that members of the *B. bifidum* species are able to exert a cooperative syntrophic effect to benefit other elements of the gut microbiota, particularly with regards to other members of (healthy) infant-associated bifidobacterial communities [52]. Notably, in vivo murine experiments involving *B. bifidum* PRL2010 showed that in contrast to other bifidobacterial strains like *B. longum* subsp. *infantis* ATCC15697 representing another member of the infant gut microbiota, PRL2010 possesses cross-feeding properties that support the growth of other bifidobacteria and exhibits a high interaction index, which measures the level of microbe–microbe interaction elicited by a bacterial strain [52]. The cross-feeding features of *B. bifidum* PRL2010 have been further analyzed by in vitro tests aimed at investigating how co-cultivation of PRL2010 cells with other strains is able to increase the growth abilities of these later as compared to growth yields obtained when these strains were cultivated independently. Remarkably, the cross-feeding characteristics of *B. bifidum* PRL2010 were evident when this strain was cultivated on host-derived glycans like mucin or HMOs [72,73] or on plant-derived carbohydrates such as starch and xylan [74]. In this context, it has been shown that the metabolism of these complex carbohydrates by PRL2010 causes the release of rather simple carbohydrates which then become accessible to other members of the (bifido)bacterial community. The trophic interaction of *B. bifidum* with other bifidobacterial species and thus its capability to sustain the growth of other bifidobacteria has recently been highlighted for *B. bifidum* ATCC 15696—notably, ATCC 15696 hydrolyzes 2’-*O*-fucosyl-lactose, a major fucosylated HMO, though it does not employ fucose that is left in the culture broth [75]. Nevertheless, fucose is a growth substrate for *Bifidobacterium breve* 24b. Notably, the release of fucose by *B. bifidum* (the donor), thereby allowing the growth of *B. breve* (the beneficiary), supports the concept of syntropy [75].

The presence of *B. bifidum* PRL2010 cells in the murine gut has been shown to provoke an expansion of the murine gut glycobiome, i.e., the overall genetic arsenal that is involved in carbohydrate metabolism, toward enzymatic degradation of plant- and host-derived glycans [52].

Another interesting activity of the members of the *B. bifidum* species in relation to their interaction with other members of the human gut microbiota, i.e., microbe–microbe cross-talk, is represented by the ability of strains of this species to produce sortase-dependent pili, particularly when residing in their natural ecological niche, the mammalian gut [30,53]. Pili also appear, apart from playing a role in host interaction as described above, to be crucial for the establishment of physical contacts with other bifidobacterial/gut commensal cells through cell aggregation [61].

## 9. Conclusions

Many *B. bifidum* strains have been described to elicit beneficial effects, including antibacterial features against pathogens like *Helicobacter pylori* [76,77], drop of apoptosis in the intestinal mucosa of premature babies affected by necrotizing enterocolitis [78], modulation of the host-immune system [79,80], and alleviation of inflammatory activities associated with certain chronic gut dysfunctions [81,82]. Nevertheless, none of these characteristics have been investigated in any detail within their natural ecological context, the infant gut. In such an environment, bifidobacterial cells are expected to drive important functions in terms of priming the immune system, enhancing the mucus layer, and modulating the establishment of a correct microbiota homeostasis. Nevertheless, in order to exploit a possible probiotic activity in the human gut, bacterial cells must be viable in this body-compartment. Furthermore, they should also colonize, compete, and persist in the human intestine. Thus, *B. bifidum* probiotic strains need to survive under gastrointestinal challenges [83] and should be able to colonize the human gut and consequently impact on the intestinal resident microbial communities. Another notable feature that is shown by human health promoting microorganisms is their ability to displace and compete with pathogens. In this context, in vitro experiments have demonstrated that *B. bifidum* PRL2010 cells strongly inhibit the adhesion of enteropathogens including *Escherichia coli* and *Cronobacter sakazakii* [83], which are frequently implicated in severe gastrointestinal diseases in infants.

Notably, for most bifidobacterial strains that are currently commercialized as probiotics, little information is available about the molecular mechanisms supporting their claimed probiotic actions.

Apart from the exploitation of *B. bifidum* strains as probiotic bacteria for infants, another possible use of these microorganisms includes the treatment of women during pregnancy. Such a probiotic administration strategy might be key in order to modulate the mother’s gut engraftment by *B. bifidum* cells prior to delivery and, therefore, to assure the establishment of an appropriate bifidobacterial community that will then be vertically inherited by the newborn. Nonetheless, various clinical testing involving *B. bifidum* must be carried out before meaningful probiotic claims (for humans) can be made.
